# Intraperitoneal Alpha-Lipoic Acid to prevent neural damage after crush injury to the rat sciatic nerve

**DOI:** 10.1186/1749-7221-4-22

**Published:** 2009-11-25

**Authors:** Mehmet Senoglu, Vedat Nacitarhan, Ergul Belge Kurutas, Nimet Senoglu, Idris Altun, Yalcin Atli, Davut Ozbag

**Affiliations:** 1 Department of Neurosurgery, Kahramanmaras Sutcu Imam University Faculty of Medicine, Kahramanmaras, Turkey; 2 Department of Physical Medicine and Rehabilitation, Kahramanmaras Sutcu Imam University Faculty of Medicine, Kahramanmaras, Turkey; 3 Department of Biochemistry, Kahramanmaras Sutcu Imam University Faculty of Medicine, Kahramanmaras, Turkey; 4 Department of Anaesthesiology and Reanimation, Kahramanmaras Sutcu Imam University Faculty of Medicine, Kahramanmaras, Turkey; 5 Department of Anatomy, Kahramanmaras Sutcu Imam University Faculty of Medicine, Kahramanmaras, Turkey

## Abstract

**Objective:**

Crush injury to the sciatic nerve causes oxidative stress. Alfa Lipoic acid (a-LA) is a neuroprotective metabolic antioxidant. This study was designed to investigate the antioxidant effects of pretreatment with a-LA on the crush injury of rat sciatic nerve.

**Methods:**

Forty rats were randomized into four groups. Group I and Group II received saline (2 ml, intraperitoneally) and a-LA (100 mg/kg, 2 ml, intraperitoneally) in the groups III and IV at the 24 and 1 hour prior to the crush injury. In groups II, III and IV, the left sciatic nerve was exposed and compressed for 60 seconds with a jeweler's forceps. In Group I (n = 10), the sciatic nerve was explored but not crushed. In all groups of rats, superoxide dismutase (SOD) and catalase (CAT) activities, as well as malondialdehyde (MDA) levels were measured in samples of sciatic nerve tissue.

**Results:**

Compared to Group I, Group II had significantly decreased tissue SOD and CAT activities and elevated MDA levels indicating crush injury (p < 0.05). In the a-LA treatment groups (groups III and IV), tissue CAT and SOD activities were significantly increased and MDA levels significantly decreased at the first hour (p < 0.05) and on the 3^rd ^day (p < 0.05). There was no significant difference between a-LA treatment groups (p > 0.05).

**Conclusion:**

A-LA administered before crush injury of the sciatic nerve showed significant protective effects against crush injury by decreasing the oxidative stress. A-LA should be considered in the treatment of peripheral nerve injuries, but further studies are needed to explain the mechanism of its neuroprotective effects.

## Introduction

The rat sciatic nerve is a well-established preparation for studying peripheral nerve injuries. Focal crush injury causes axonal interruption but preserves the connective sheaths (axonotmesis). As regards this type of injury, nerve regeneration is usually successful [1].

The increased formation of reactive oxygen species (ROS) and decreased antioxidant defense is defined as oxidative stress, which is widely recognized as an important feature of many diseases. Superoxide dismutase (SOD), and catalase (CAT) are cellular antioxidants, which protect cells from oxidative stress. Lipid peroxidation (LPO) is one of the most important expressions of oxidative stress induced by ROS. Malondialdehyde (MDA) is an indicator of lipid peroxidation, and increases in various diseases [2].

Alpha-Lipoic acid (a-LA) is a powerful lipophilic antioxidant in vitro and in vivo, which plays a pivotal role as cofactor in many mitochondrial reactions, readily absorbed from the diet and can easily cross the blood brain barrier [3].

It is known to act as scavenger of many reactive oxygen species and to interact with other antioxidants such vitamin C and vitamin E, resulting in their regeneration. Due to its antioxidant activity, a-LA has been proposed as a treatment for oxidative disorders of the nervous system that involve free radicals since it exerts a profound neuroprotective effect in experimental models of stroke, trauma, degenerative disorders of the CNS and diabetes [3].

Administration of a-LA to rodents has been demonstrated to reduce the damage that occurs after ischemia-reperfusion injuries in the cerebral cortex [3], heart [4,5] and peripheral nerve [6], and after injection of NMDA into the striatum [7]. However, to our knowledge, the effects of a-LA on crush injury have not been investigated in the English literature [3-7].

The increased formation of ROS and decreased antioxidant defense is defined as oxidative stress, which is widely recognized as an important feature of many diseases. SOD, and CAT are cellular antioxidants, which protect cells from oxidative stress. LPO is one of the most important expressions of oxidative stress induced by ROS. MDA is an indicator of lipid peroxidation, and increases in various diseases [2].

The purpose of this study was to investigate the effects of a-LA on sciatic nerve injury by measurement of SOD and CAT activities, as well as MDA level in sciatic nerve crush injury model in rats.

## Materials and methods

### Animals and Surgery

This prospective, experimental, sham-control study was performed in the animal laboratory of the Kahramanmaras Sutcu Imam University, Faculty of Medicine. Female Sprague-Dawley rats were obtained from Experimental Research Laboratory of Sutcu Imam University Faculty of Medicine. The experimental design was approved by the Ethics committee of KSU. Rats were fed with standard rat diet routinely, however they were deprived of food for 12 h prior to the first operation. All rats had free access to standard rat chow and tap water.

Forty adult female Sprague-Dawley rats (200-250 grams) were used in this study. Rats were randomly divided into four groups including one sham, one control and two treatment groups.

**Group I **- (Sham group) Normal adult female rats (Non-crush): Non-crush group, no intervention was made, simply sciatic nerve samples were taken.

**Group II **- (Control group) 60 seconds of sciatic crush was performed and then sciatic nerve samples were taken at the 1st hour.

**Group III **- Crush-a-LA group (1 hr): 100 mg/kg intraperitoneal a-LA injection was done 24 and 1 hour before crush injury. Sixty seconds of crush was performed. Sciatic nerve samples were taken at the 1st hour.

**Group IV **- Crush-a-LA group (3rd day): 100 mg/kg intraperitoneal a-LA injection was done 24 and 1 hour before crush injury. Sixty seconds of crush was performed. Sciatic nerve samples were taken on the 3rd day.

Groups I and II received saline (2 ml, intraperitoneally). However, groups III and IV received saline plus a-LA (100 mg/kg, 2 ml, intraperitoneally [MEDA Pharma GmbH & Co. KG]) [8] at 1 h and 24 h before the crush injury. We aimed to investigate the early effects of antioxidant therapy with a-LA. In Group I, sciatic nerve was explorated but not crushed. Sciatic nerve injury was induced in groups II, III and IV. Briefly stated, exploration was conducted under anesthesia with intraperitoneal pentobarbital (50 mg/kg), while body temperature was maintained by using a heating blanket at 35-37°C. The sciatic nerve was exposed in the mid-gluteal region through biceps muscle dissection under an operating microscope, crushed by a #4 Jeweler's forceps at the mid-point for 60 seconds, then unclamped. The site of crush was marked with a 5-0 suture tied in surrounding muscle. The operated animals were allowed and survived. The nerves were re-exposed under the operating microscope one hour later in groups I, II and III, and 3 days later in Group IV and the nerve tissue was harvested. One-centimeter-long sciatic nerve segments centered on the lesion site points were collected for biochemical analyses. No prophylactic antibiotics were given. The experimental model was very well tolerated. No animal died during the operations.

### Preparation of tissue homogenates

Tissue samples were immediately excised, weighed, perfused with 1.15% ice-cold KCl, minced, then homogenized in five volumes (w/v) of the same solution, using a Heidolph 50110 R2R0 homogenizer. Antioxidant enzymes and MDA assays were performed on the supernatant preparation in a Sorvall RC-2B centrifugation of the homogenate at 14.000 rpm for 30 min at +4°C.

### Evaluation of biochemical parameters

CAT activities were determined by measuring the decrease in hydrogen peroxide concentration at 230 nm by the method of Beutler [9]. Assay medium consisted of 1 M Tris HCl-5 mM Na_2_EDTA buffer solution (pH 8.0), 1.0 M phosphate buffer solution (pH 7.0), and 10 mM H_2_O_2_. CAT activity was expressed as U/mg protein.

SOD activity was measured according to the method described by Fridovich [10]. This method employs xanthine and xanthine oxidase to generate superoxide radicals which react with p-iodonitrotetrazolium violet (INT) to form a red formazan dye which was measured at 505 nm. Assay medium consisted of the 0.01 M phosphate buffer, CAPS (3-cyclohexilamino-1-propanesulfonicacid) buffer solution (50 mM CAPS, 0.94 mM EDTA, saturated NaOH) with pH 10.2, solution of substrate (0.05 mM xanthine, 0.025 mM INT) and 80 U/L xanthine oxidase. SOD activity was expressed as U/mg protein.

LPO level in the tissue samples was expressed as MDA. It was measured according to procedure of Ohkawa et al [11]. The reaction mixture contained 0.1 ml of sample, 0.2 ml of 8.1% sodium dodecyl sulphate (SDS), 1.5 ml of 20% acetic acid and 1.5 ml of 0.8% aqueous solution of TBA. The mixture pH was adjusted to 3.5 and volume was finally made up to 4.0 ml with distilled water and 5.0 ml of the mixture of n-butanol and pyridine (15:1, *v/v*) were added. The mixture was shaken vigorously. After centrifugation at 4000 rpm for 10 min, the absorbance of the organic layer was measured at 532 nm. The results of MDA were expressed as nmol/mg protein.

### Assay of Protein levels

The protein concentration of the tissue was measured in digital Spectronic-20 spectrophotometer by the method of Lowry [12].

### Statistical analysis

All variables were expressed as medians, mean ± standard deviation with the range. Data were analyzed using Mann-Whitney *U*-test. Differences were considered significant when the probability was less than 0.05. All data were entered and processed by an SPSS 9.05 for Windows statistical package.

## Results

**Figure 1:**
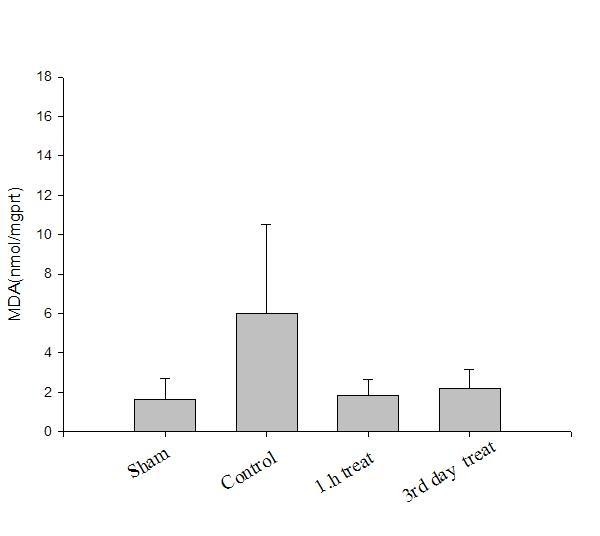
**Sciatic nerve MDA (nmol/mg protein) levels in all groups**.

**Figure 2:**
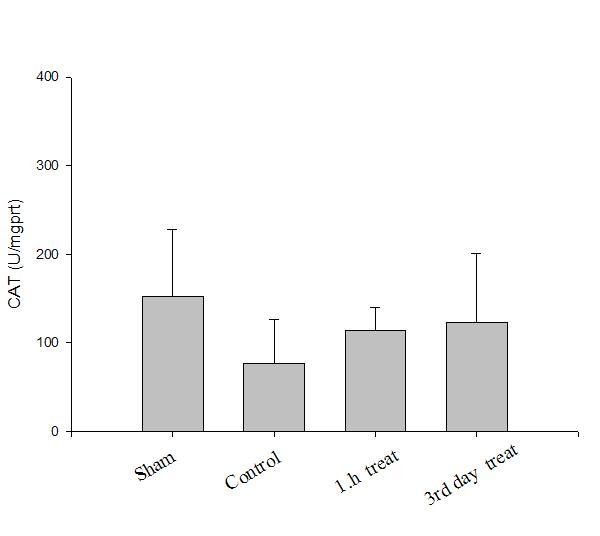
**Sciatic nerve CAT (U/mg protein) levels in all groups**.

**Figure 3:**
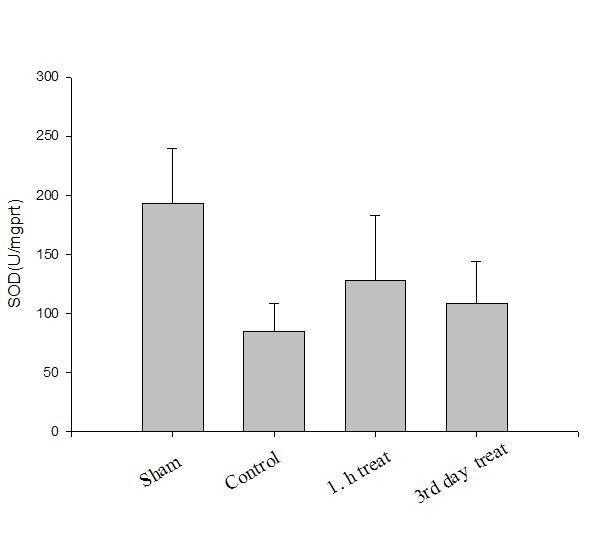
**Sciatic nerve SOD (U/mg protein) levels in all groups**.

Oxidative parameter results are presented in Table 1. Results of the antioxidants levels in all groups are presented in Figures 1, 2, 3.

**Table 1:** The activities of antioxidant enzymes and MDA levels in four groups.

	MDA (nmol/mg prt)		CAT (U/mg prt)		SOD (U/mg prt)	
	
	Mean ± SD	Median (min-max)	Mean ± SD	Median (min-max)	Mean ± SD	Median (min-max)
Group I (sham)	1.64 ± 1.05	1.49 (0.48-3.90)	152.18 ± 75.29	150.30 (55.27-339.72)	193.21 ± 46.38	194.60 (111.06-276.40)

Group II (control)	6.02 ± 4.50	4,10 (3.25-16.80)	76.43 ± 50.43	64.57 (24.38-157.75)	84.58 ± 23.83	79,00 (50.86-121.96)

Group III Crush-a-LA group (1 hr)	1.84 ± 0.83	1,90 (0.25-3.39)	114.14 ± 25.99	112.81 (73.49-151.86)	128.12 ± 54.60	125.82 (70.45-217.56)

Group IV Crush-a-LA group (3rd day)	2.18 ± 0.98	2.12 (0.60-3.90)	122.89 ± 77.63	98.82 (76.21-339.72)	108.21 ± 35.56	103.93 (54.40-172.01)

Values were expressed as mean ± SD and median and range. Group I (Control), Group II (Sham), Group III and IV (Treatment).

Compared with sham group (Group I), tissue SOD and CAT activities decreased and MDA level elevated significantly in the control group (Group II), which indicated crush injury (p < 0.05). After a-LA treatment tissue SOD and CAT activities increased and MDA level decreased significantly, at the first hour (Group III, p < 0.05) and on the 3^rd ^day (Group IV, p < 0.05). However, there were no significant differences between treatment groups (Groups III and IV, p > 0.05).

## Discussion

In the present study, we investigated antioxidant effects of a-LA on sciatic nerve which was subjected to 60 seconds of crush injury, based on differences observed in biochemical parameters measured. We observed that a-LA had antioxidant effects on injured sciatic nerve and these effects were similar at the first hour and on the 3^rd ^day.

The pathophysiology of the crush injury has not been fully understood, and it has been debated whether the ischemia, secondary to compression, or the mechanical deformation of nerve fibers per se is the more significant etiologic factor [13].

Nerve injury may depend on the length of time of crush insult. During a pilot study of our research, we showed that especially 60-second compression caused injury in sciatic nerve. After the injury due to the tissue destruction, free oxygen radicals increase and cause tissue damage [14,15].

Normal cell functions and integrity of cell structures may be broken via considerable reactivity of ROS. The organism has enzymatic (e.g. superoxide dismutase, catalase, glutathione peroxidase) and non-enzymatic (e.g. vitamin C, vitamin E) antioxidant mechanisms that work as scavengers for the harmful ROS. Radical-scavenging antioxidants are consumed by the increased free radical activity. Oxidative stress can be defined as an increase in oxidants and/or a decrease in antioxidant capacity. Although determination of either oxidants or antioxidant components alone may give information about the oxidative stress, determination of oxidants along with antioxidants is more useful in this context. Therefore, oxidants and antioxidant capacity should be measured simultaneously to assess oxidative stress more accurately. In addition, the total plasma LPO level, as an indicator of oxidative stress, reflects the redox balance between oxidation and anti-oxidation. In addition, excess amounts of ROS generated in inflamed tissues can cause injury to host cells and also induce DNA damage and mutations [16]. And, oxidative DNA damage has been suggested to play an important role in the development of cancer [17]. In several studies, increase in oxidative components or decrease in antioxidants or both have been reported in subjects with either acute or chronic various diseases [18-21].

Antioxidant activity is a relative concept: it depends on the kind of oxidative stress and the kind of oxidizable substrate (e.g., DNA, lipid, protein). To control oxidative processes, biological systems have been equipped with several antioxidant mechanisms. Antioxidant enzymes such as SOD and CAT are concerned with the removal of superoxide anion and peroxide. An imbalance between oxidative and antioxidative processes results in oxidative stress. Drugs can intervene in oxidative processes as antioxidants and delay or prevent their damaging effects. A-LA acid is an example of an existing drug therapeutic effect of which has been related to its antioxidant activity. Many experimental results show that both lipoic acid and Dihydrolipoic acid (DHLA) can improve the antioxidant capacity of tissue against different forms of oxidative stress. Hence, some physicians started to administer lipoic acid to patients with liver cirrhosis, mushroom poisoning, heavy metal intoxication and diabetic polyneuropathy [22].

The SOD-CAT system provides the first defense against oxygen toxicity. SOD catalyzes the dismutation of the superoxide anion radical to water and hydrogen peroxide, which is detoxified by the CAT activity. Usually a simultaneous induction response in the activities of SOD and CAT is observed when an exogen antioxidant is applied [23]. In the present study, the activities of SOD and CAT were also found to be high in sciatic tissue of rats in groups III and IV. The activity of SOD was reported to be higher in various diseases by several workers [19,21] indicating high production of superoxide anion radical. Since CAT levels were detected high in sciatic tissue of rats in groups III and IV, this may be attributed to high production of peroxide radicals. Increased SOD and CAT activities in in those groups may be a response against oxidative stress.

The extent of LPO is determined by the balance between the production of oxidants and the removal and scavenging of those oxidants by antioxidants. Therefore, lipid peroxidation has been extensively used as a marker of oxidative stress [24]. Antioxidants are potential candidates for prevention or treatment of oxidative damage and free radical injury [5,25].

In this study, tissue MDA levels increased and CAT and SOD activities decreased significantly in the control group compared with sham group showing crush injury. After a-LA treatment, groups III and IV had significantly higher tissue SOD and CAT activities and lower MDA levels than control group showing antioxidative effects. However, these antioxidant effects were similar in treatment groups (groups III and IV) showing that preventive antioxidant effects of a-LA took place in the early phase. This finding was concordant with the finding of decreased oxidative injury (i.e. decreased MDA levels in nerve tissue) seen in pretreated groups, which was confirmed by biochemical parameters. Oxidative stress is a mechanism of nerve injury but likely not the major mechanism, and that therapeutic strategy for neuroprotection from crush injury should not be based on antioxidants alone.

Further clinical and laboratory investigations focusing on specific mechanism of antioxidant effects of a-LA, and its therapeutic effects in peripheral nerve injury is warranted.

## Abbreviations

SOD: superoxide dismutase; CAT: catalase; MDA: malondialdehyde; a-LA: Alfa Lipoic acid; CNS: Central Nervous System; ROS: reactive oxygen species; LPO: Lipid peroxidation; DHLA: Dihydrolipoic acid.

## Competing interests

The authors declare that they have no competing interests.

## Authors' contributions

MS and NS designed the study and drafted the manuscript. MS and IA performed experimental operations. EBK, YA and DO had specimen collection of this experimental study. VN and performed the statistical analysis. All authors read and approved the final manuscript.
